# Fifty Years of Clinical Application of Newcastle Disease Virus: Time to Celebrate!

**DOI:** 10.3390/biomedicines4030016

**Published:** 2016-07-20

**Authors:** Volker Schirrmacher

**Affiliations:** Immunological and Oncological Center (IOZK), Tumor Immunology, 50674 Cologne, Germany; V.Schirrmacher@web.de; Tel.: +49-(0)221-420-399-25

**Keywords:** oncolysis, immunogenic cell death, dendritic cells, tumor vaccine, immune activation, virotherapy, immunotherapy

## Abstract

This review provides an overview of 50 years of basic and clinical research on an oncolytic avian virus, Newcastle Disease Virus (NDV), which has particular anti-neoplastic and immune stimulatory properties. Of special interest is the fact that this biological agent induces immunogenic cell death and systemic anti-tumor immunity. Furthermore, localized oncolytic virotherapy with NDV was shown to overcome systemic tumor resistance to immune checkpoint blockade immunotherapy. Clinical experience attests to low side effects and a high safety profile. This is due among others to the strong virus-induced type I interferon response. Other viral characteristics are lack of interaction with host cell DNA, lack of genetic recombination and independence of virus replication from cell proliferation. In this millennium, new recombinant strains of viruses are being produced with improved therapeutic properties. Clinical applications include single case observations, case series studies and Phase I to III studies.

## 1. Introduction

Progress in science and medicine is often driven by new observations or ideas and the development of new concepts and strategies. Sometimes it takes decades before one can say whether such a new idea was right and whether the concept has a lasting effect. This will be exemplified by the concept of oncolytic viruses and post-oncolytic immunity.

The first report [1] of results using NDV as a treatment for human cancer (a patient with acute leukemia) was published in 1964. Important new concepts were developed by Lindenmann, a Swiss virologist and immunologist who, together with the British virologist Isaacs, described for the first time type I interferon (IFN) and its interference with virus replication [2]. Lindenmann also described viruses as immunological adjuvants in cancer [3].

## 2. Treatment of Cancer Patients by NDV (Newcastle Disease Virus) Oncolysate Vaccines

The 1960s were devoted to oncolytic viruses and later to research on post-oncolytic anti-tumor immunity. All kinds of viruses were found and studied but only few remained of further interest. Among these few was the avian paramyxovirus NDV. By that time, attenuated strains of NDV had been used for almost two decades to prevent Newcastle disease in birds. The inability of this virus to cause serious illness in humans had been established and non-lytic, as well as oncolytic, strains were described [4]. It was Cassel from Atlanta (GA, USA), together with Garrett, who in 1965 reported on NDV (*oncolytic strain 73 T*) as an antineoplastic agent [5]. When, thereafter, they observed the development of tumor immunity after viral oncolysis [6] they focused on the development of viral oncolysates as vaccines for post-operative management of malignant melanoma patients. Together with Murray, Cassel selected the most suitable melanoma cell lines and established a protocol for the production of a standardized NDV melanoma oncolysate vaccine. Vaccinations were performed over many years and have never stopped completely [7].

Two Phase II clinical studies involving 32 and 51 patients were conducted successively in Stage II metastatic melanoma (AJCC stage III). In these studies, NDV oncolysate was examined as an adjunctive immunotherapeutic agent. A ten-year follow up of these 83 treated patients revealed that over 60% were alive and free of recurrent disease [8]. At 15-years follow-up, 55% were alive. Additionally, alterations in the CD8 T cell repertoire were reported [9]. Older studies in the US reported postsurgical survival figures for Stage II cases of 5%–15%. More contemporary studies indicate a 33% survival at 10 years. By including other findings, Cassel concluded as follows: “The unusual disease-free survival periods in the present study, including exceptional survivals in 21 patients with head and neck disease and six cases with cerebral metastases, suggest a unique role for the administration of NDV oncolysate in the management of Stage II malignant melanoma patients [8]”.

A similar Phase II study of NDV oncolysates has been conducted in Germany by Kirchner, Anton and Atzpodien, involving 208 patients with locally advanced renal cell carcinoma [10]. NDV strain *73 T* was used to prepare autologous oncolysates that were given to patients by subcutaneous injection once a week for 8 to 10 weeks, beginning one to three months after radical surgery (nephrectomy and regional lymph node dissection). Two cytokines, low-dose recombinant interleukin-2 and recombinant interferon-α were added to the oncolysate vaccines. The researchers concluded that the results demonstrated improved disease-free survival (DSF) in comparison with survival data published for similar patients who were treated by surgery alone.

## 3. Treatment of Cancer Patients by Oncolytic NDV

A Hungarian research effort led by Csatary advocated the use of an attenuated oncolytic veterinary vaccine strain, which they termed *MTH-68/H*. One placebo-controlled Phase II clinical trial [11] included 33 patients in the NDV treatment group and 26 patients in the placebo group. The patients had various advanced chemorefractory cancers. The route of virus application was interesting and new: The virus was applied via inhalation in order to target lung metastases. NDV (4000 U/day) was inhaled twice weekly for a period of six months. The high virus doses were well tolerated and the clinical results, although not randomized, suggested a decrease in cancer-related symptoms and better survival. The two-year survival rates were 21% in the NDV treatment group and 0% in the placebo group [11].

A selected case series study of four high-grade gliomas treated with *MTH-68/H* reported on radiographically-documented responses and long survival with improved symptomatology [12].

In Israel, another NDV strain (*HUJ*, lentogenic) was administered intravenously to 14 glioblastoma multiforme (GBM) patients using intra-patient dose escalation (1–11 billion infectious units) followed by three cycles of 55 billion infectious units. Toxicity was minimal and a maximal tolerated dose was not achieved. One patient achieved a complete response, although of only transient duration (about three months), while the others developed progressive disease [13].

In a Phase I trial conducted in the United States by Wellstat Biologics, another lytic NDV strain, *PV701*, was tested in patients with advanced cancers. This strain had been extensively tested in pre-clinical studies in vitro [14,15] and in human tumor xenotransplanted mice [16]. Seventynine patients, whose tumors had not responded to conventional therapy, were given escalating doses of virus intravenously. Doses of three billion infectious particles were well tolerated. Dose-limiting toxicities included dyspnea, diarrhea and dehydration. In some patients a transient thrombocytopenia and diffuse vascular leak was observed [17]. When patients were desensitized with a lower initial dose, the maximal tolerated dose was increased 10-fold [18].

## 4. Treatment of Cancer Patients by Autologous Tumor Cell Vaccine ATV-NDV

In the 1980s, my group at the German Cancer Research Center in Heidelberg, had established an animal model system to study cancer metastasis and immunotherapy. Upon intradermal transfer of leukemic ESb tumor cells (a highly aggressive variant from the parental line Eb) to syngeneic mice, a local palpable tumor developed within 10 days. To simulate a model system for post-operative immunotherapy, we removed the primary tumor and tried to develop a vaccine against the remaining micrometastatic disease (mainly in the liver).

Our concept was to infect tumor cells by a virus to make it more immunogenic and to develop thereof an irradiated virus-infected live cell tumor vaccine. From Lindenmann (Zürich, Switzerland) we obtained human influenza virus and a mouse strain resistant to this virus. Although we saw occasionally anti-tumor effects in vivo, reproducibility was difficult. Additionally, the virus had to be adapted to the murine tumor cells first and the virus-resistant strain of mice had to be crossed with the strain from which the tumor line arose to be able to perform respective in vivo experiments. There were also safety aspects to be dealt with concerning the laboratory personnel.

All of this changed in 1984, when we got ahold of another virus, namely the avian paramyxovirus NDV. The strain used (*Ulster*) is lentogenic and not oncolytic so that infected tumor cells remain viable and immunogenic, at least within a period of 24–48 h. In contrast to influenza virus, NDV could infect ESb cells without adaptation.

The concept of using a live cell vaccine rather than a lysate was based on observations by Kobayashi (Chiba, Japan) who had developed the term “tumor xenogenization”, and had described the higher immunogenicity of live cell vaccines compared to lysates [19].

In our first post-operative anti-tumor vaccination experiment, we applied i.p. vaccines consisting of 10 million irradiated ESb tumor cells, which had been infected with increasing doses of NDV (*Ulster*). In the control group, which was operated and vaccinated with ESb vaccine without NDV, all animals died within three weeks. In contrast, in one group, which was vaccinated with ESb cells infected by a low dose of virus, more than 50% of the mice survived long-term [20].

Until the end of the 1980s, we were busy with pre-clinical studies in various other animal tumor models to find out whether similar effects could be seen. This was found to be the case. Thus, we concluded that a live cell tumor vaccine modified by a low dose of NDV could in general induce protective anti-tumor immunity. This was found true for the ESb lymphoma of DBA/2 mice, for the B16 melanoma of C57BL/6 mice and also for the L10 lung carcinoma of guinea pigs. The protective immunity induced in syngeneic animals against their tumor lines turned out to be highly specific for the individual tumor. There was no cross-reactivity even with the parental line upon re-challenge of pre-vaccinated mice [21]. Further studies revealed that vaccination augmented tumor-specific cytotoxic T cell (CTL) responses [22] and tumor-specific T helper responses [23]. Potentiation of the CTL responses involved the induction of interferon-α/β [24] and was a result of CD4 and CD8 immune T cell cooperation [23]. The vaccine that we had developed was termed ATV-NDV. This stands for Autologous Tumor cell Vaccine modified by infection with NDV.

### 4.1. From Mouse to Man: Translational Research in the 1990s

The decision to go further from mouse to man was based on two considerations: (i) the urgent need in the clinic for a new post-operative strategy against metastases and (ii) the development in the USA of an autologous live cell tumor vaccine mixed with Bacillus Calmette Guérin (BCG) by Hanna and colleagues [25,26].

Of course, there were a number of questions to be solved: Would human tumor cells also be infectable by non-lytic NDV? How could one best isolate tumor cells from a fresh operation sample without losing cell viability? How should one inactivate tumor cells? What dose and where to apply? What type of tumor should be tried first?

Like us in Heidelberg, the group of Hanna had based their vaccine on careful pre-clinical post-operative vaccination studies in animals [24]. The term used for this type of immunotherapy worldwide was “active-specific immunotherapy (ASI)” because it consisted of an active immunization procedure in cancer patients and the product was specific from components of the patient`s tumor cells. At this point, it is perhaps important to realize that in those early days many things were not known yet, neither the nature of the T cell receptor nor that of tumor-associated antigens (TAAs). There were hot disputes as to whether TAAs existed at all, and whether or not they were also expressed on human tumor cells.

After a series of tests of human tumor cell lines or of tumor cells from fresh operation specimens we reported that human tumor cell modification by virus infection is an efficient and safe way to produce cancer vaccine with pleiotropic immune stimulatory properties when using NDV [27].

### 4.2. Clinical Studies with ATV-NDV

A Phase I clinical trial in patients with colorectal carcinoma (CRC) helped to establish the optimal number of tumor cells and the optimal amount of virus to use to produce the best possible immune response. Immune responses were monitored by means of a skin delayed-type hypersensitivity (DTH) test using as intra-dermal challenge either irradiated ATV-NDV vaccine or irradiated ATV cells. In one study, 12 of 16 (75%) patients showed increased immune system reactivity against uninfected autologous tumor cells after vaccinations [28].

Post-operative vaccinations were performed three times in two-week intervals. This appeared sufficient to obtain peak anti-tumor DTH reactivity. This means three rounds of expansion and retraction of tumor-reactive T cells involving three waves of memory T cells changing between a status of activated effector memory (EMT) T cells in peripheral tissues and of resting central memory (CMT) T cells in the bone marrow [29].

One clinical study evaluated the effect of vaccine quality on the survival of patients treated with ATV-NDV. In this retrospective study, survival was estimated separately for three groups of patients who had early breast cancer (*n* = 63), metastatic breast cancer (*n* = 27) or metastatic ovarian cancer (*n* = 31) and who had sufficient numbers of recovered tumor cells to allow at least two vaccinations. The ATV-NDV vaccine was classified as either high quality or low quality on the basis of the following parameters: the number of tumor cells and the percentage of live tumor cells. Overall survival (OS), four years after surgery, was estimated to be 96% for the patients with early breast cancer who had received a high quality vaccine (*n* = 32) compared with an OS of 68% for those who had received a low-quality vaccine (*n* = 31), a difference that was statistically significant [30].

Results from a pilot trial of ATV-NDV application in 20 patients with stage III and IV head and neck squamous cell carcinomas (HNSCC) suggest that the vaccination strategy can stimulate human antitumor immune responses in a manner similar to those found in animal models, and may significantly prolong five-year survival rates in this patient population. The study demonstrated the feasibility and safety of the vaccine regimen, and no major side effects were observed in any of the patients [31].

A Phase II trial involved patients with locally advanced CRC (*n* = 57). Forty-eight patients were treated with ATV-NDV and nine were treated with autologous tumor cell vaccine (ATV) mixed with BCG bacteria, the adjuvant used by MG Hanna and colleagues in their studies in the USA. Two years after surgery, OS for patients treated by ATV-NDV was 98% compared with 67% for the patients treated with ATV + BCG and to 74% for historical control subjects [32].

Another Phase II trial involved renal cell cancer patients (*n* = 40), whose disease had spread from the kidneys to at least one other organ. After radical nephrectomy the patients were treated by ATV-NDV at three and five weeks after surgery. They were also given subcutaneous injections of low-dose recombinant interleukin-2 and recombinant IFN-α. Five patients had a complete response and six a partial response. After four years of follow-up, OS for these 11 patients was 100%. Among the remaining 29 patients, 12 had stable disease (median survival = 31 months) and 17 had progressive disease (median survival = 14 months) [33].

A further Phase II study was performed with patients suffering from GBM. They were vaccinated with ATV-NDV vaccine obtained from cell culture. The objective was to assess feasibility, safety and clinical benefit. The median progression-free survival of vaccinated patients (*n* = 23) was 40 weeks versus 26 weeks in 87 non-vaccinated control subjects from the same time period and the same clinic. The median OS was 100 weeks (vs. 49 weeks in control subjects, *p* < 0.001). In the vaccinated group, immune monitoring revealed significant increases of skin DTH reactivity, of numbers of tumor-reactive MTCs in the blood and in the number of CD8+ tumor-infiltrating T lymphocytes in frozen tissue slices from GBM recurrences. There was one complete remission of non-resectable remaining brain tumor [34].

A prospectively randomized Phase II/III trial investigated the efficiency of ATV-NDV after liver resection for hepatic metastases of CRC as a tertiary prevention method. Twenty-five of such stage IV CRC patients were vaccinated and compared with a similar number of non-vaccinated control patients. After an exceptionally long follow-up period of 9–10 years, there was no significant difference between the vaccinated and the control arm. However, when stratified for tumor localization there were significant differences between vaccinated colon and rectum carcinoma patients. While there was no significant difference between control and vaccinated rectal cancer, a significant benefit was seen in the colon cancer subgroup in terms of long-term metastasis-free survival and OS. In the control arm, 78.6% had died, and, in the vaccinated arm, only 30.8%. The trial provides clinical evidence for the value and potential of the ATV-NDV cancer vaccine [35,36].

## 5. Treatment of Cancer Patients by ATV-NDV with Attached Bispecific Antibody αHNxαCD28

While most of the clinical studies with ATV-NDV showed promising results, there was still a majority of patients that had to be considered as non-responders. Thus, efforts were undertaken to further increase the effectiveness of the vaccine.

One strategy consisted of adding NDV-specific single chain antibodies with dual specificity (bispecific scFv antibodies, bsAb) for attachment to ATV-NDV to augment T-cell stimulatory signals and thereby overcome potential T cell anergy [37].

In a phase I clinical study, 14 CRC patients with late-stage disease which could not anymore be subjected to surgery with curative intent were treated with ATV-NDV-bsHN-CD28. No severe adverse events were recorded. While before vaccination none of the patients had detectable levels of tumor-reactive blood circulatory T cells, all patients showed an immunological response of tumor-reactive T cells, at least once during the course of five vaccinations. Additionally, there was a dose-response relationship with the costimulatory molecule attached to the vaccine. A partial response of metastases was documented in four patients. The study suggests that the second-generation three-component vaccine is safe and can reactivate possibly anergic T cells from a chronic disease like advanced-stage cancer.

## 6. Treatment of Cancer Patients by Autologous Viral Oncolysate-Pulsed DC (VOL-DC)

The other strategy was to combine ATV-NDV with dendritic cells (DCs) in order to improve the de novo generation of TAA-specific cells from naïve T cells. A protocol for the generation of viral oncolysate-pulsed DCs (VOL-DCs) has been developed by the Immunological and Oncological Center (IOZK) in Cologne (Germany). In 2015, IOZK received an official permit for an individual application of this product to cancer patients. This permit required, among other factors, the production of high-quality oncolytic NDV under Good Manufacturing Procedure (GMP) guidelines. This was, worldwide, the first time that this had been achieved with NDV.

For further information of the strategy which has been developed by IOZK to treat cancer patients, we refer to two recent case reports.

One demonstrates long-term remission of prostate cancer with extensive bone metastases. The patient had failed standard therapy, but then achieved complete remission following combined treatment with local hyperthermia (LHT), systemic NDV and vaccination with VOL-DC. The treatment induced a long-lasting antitumor memory T-cell response [38].

The other case report relates to long-term survival of a breast cancer patient with extensive liver metastases. After operation the patient refused further standard therapy. Instead, she was treated with LHT, systemic NDV and VOL-DC. A continuous high quality of life was reported, and the patient survived more than 66 months after the initial diagnosis. No recurrence of further metastases developed under treatment. Following treatment, a long-lasting tumor-reactive memory T-cell responsiveness was documented. This possibly explains the favorable course of disease. Since this combination of therapies is not restricted to a particular tumor type, further exploration is warranted.

## 7. Results from China

In 2003, Liang et al. from China reported results from applications of autologous tumor cell vaccine and NDV vaccine (strain *La Sota)* in the treatment of tumors of the digestive tract [39]. Details about the tumor vaccine are lacking, most likely it consisted of formalin-fixed and/or paraffin-embedded tumor tissue, a type of vaccine developed in China and India. Tumor vaccine and NDV vaccine were applied separately but the article does not provide more detailed information. Three hundred and ten patients, suffering from Gastrointestinal Carcinoma (stage I–IV), were treated and compared to 257 patients in which resection was performed without further vaccination. A significantly improved mean and median survival was calculated for the vaccination group (7 years versus 4.46 years) [39]. The high number of patients treated is remarkable. However, neither concurrent therapy, nor level of evidence score, are mentioned.

Hepatocellular Carcinoma (HCC) is a cancer of major significance in China. Bian worked from 2003 to 2006 in my division at the German Cancer Research Center in Heidelberg. Back in China, she focuses on the development of new strategies for fighting this disease. We had developed a bispecific adaptor protein (αHN-IL-2) binding to NDV and to the interleukin-2 receptor IL-2R. The latter could be used as a surrogate tumor-associated target. The aim of her project in Heidelberg was: (i) to specifically target tumor tissue by oncolytic NDV; (ii) to improve the delivery system for systemic application of NDV via the bispecific adapter protein; and (iii) to investigate anti-tumor activity and side effects. Tumor therapy experiments in mice showed that the side-effects of high-dose virotherapy were greatly reduced by the adapter protein, and that the anti-tumor effects were mostly undiminished [40]. This study involved the use of the velogenic oncolytic strain *Italien*.

Bian and colleagues managed to construct a reverse genetics system including a minigenome rescue [41] for NDV *Italien* and generated two recombinant NDVs, carrying a marker gene encoding either enhanced green fluorescent protein (EGFP) or firefly luciferase [42]. Recently, this research group provided a new strategy of arming oncolytic NDV with therapeutic antibody to enhance anti-tumor efficacy in orthotopic hepatoma-bearing mice. They created a new recombinant strain (*rNDV-18HL*) with an incorporated antibody gene (cHAb18). This chimeric antibody is directed against tumor-associated antigen CD147 and demonstrated the inhibition of invasion and migration of HCC cells. The new antibody, expressing virus, selectively replicated in orthotopic HCC xenografts leading to antibody expression in situ. It induced tumor necrosis, reduced the intrahepatic metastases, and prolonged the survival in mice [43].

Another finding from this group is of no less importance. Activated hepatic stellate cells (HSCs) are the crucial factor responsible for liver fibrosis and involved in development of HCC by interaction with tumor cells. It was reported in 2009 that NDV can repress the activation of human hepatic stellate cells and can revert the development of hepatic fibrosis in mice [44].

A recent interesting report from China relates to the incorporation into NDV of a suicide gene coding for cytosine deaminase (CD). In animal experiments, the application of 5-FC significantly enhanced cell death of HepG2 tumor cells in vitro and increased survival of H22 tumor-bearing mice [45].

Apart from Bian there were other women scientists from China in my Division who performed excellent and important work with NDV. Bai who, in China, had already done her thesis work on NDV, demonstrated that DCs pulsed with viral oncolysates potently stimulate autologous T cells from cancer patients [46]. Zeng identified via cDNA transfectants viral HN as a pathogen-associated molecular pattern (PAMP) inducing strong type I IFN responses [47]. Ni demonstrated that the HN of NDV is a powerful molecular adjuvant for DNA anti-tumor vaccination [48].

## 8. Special Characteristics of NDV and Other Paramyxoviruses

Advantages exist in using paramyxoviruses as oncolytic agents versus representatives of other viral families (e.g., retroviruses or DNA viruses).

### 8.1. High Safety Profile

**Lack of gene exchange via recombination**. Phylogenetic analyses of the *F*, *HN* and *M* genes from NDV isolates of the last 50 years demonstrate the existence of distinct lines [49,50]. These arose, most likely, by accumulation of point mutations. There was not a single case of gene exchange via recombination.

**Lack of interaction with host cell DNA**. The cytoplasmic replication results in a lack of host genome integration and recombination. The cytoplasmic replication makes the virus independent of host cell DNA replication.

**Virus replication independent of cell proliferation**. This is in contrast to chemotherapy (CT) and radiotherapy (RT) which require target cells to be in a proliferating state. Many tumor cells in a resting state, such as tumor stem cells or cells from tumor dormancy, may not be affected by CT or RT, but are likely to become targeted by NDV.

**Mild side effects in cancer patients**. The reported side effects after experience with clinical application for 50 years are grade 1 and 2, with the most common being mild fever.

### 8.2. Tumor-Selective Replication and Oncolysis

The list of oncolytic paramyxovirus representatives includes attenuated *Measles virus* (MV), *Mumps virus* (MuV), *Sendai virus* (SeV) and NDV. Metastatic cancer cells frequently overexpress on their surface molecules that can serve as virus receptors. For NDV, cell surface receptors are sialylated glycoproteins or glycolipids recognized by the cell adhesion domain of HN. Individual strains of NDV are classified as lytic or nonlytic viruses. Both types of viruses can kill cancer cells, but lytic strains have the potential to do this more quickly because they damage the plasma membrane of infected cells. In addition, lytic strains show multicyclic replication while nonlytic viruses show only monocyclic replication [51]. Nonlytic strains interfere with cell metabolism: The competition between virus and host cell for the protein synthesis machinery causes an ER stress response, which is associated with phosphorylation of PERK and eIF2α [52], which then shuts off protein synthesis.

Paramyxoviruses are capable of inducing syncytium-mediated lysis of cancer cells. Virus release is facilitated by the viral HN-associated neuraminidase and syncytium formation by the viral F protein. This process allows progeny viruses to spread within tumor tissue. In the case of nonlytic strains, progeny virus particles are non-infectious due to an uncleaved F protein, while lytic viruses express a cleaved F protein and release infectious virus progenies.

NDV-induced apoptosis is dependent on upregulation of TNF-related apoptosis-inducing ligand (TRAIL) and caspase activation. The virus mediates its oncolytic effect by both intrinsic and extrinsic caspase-dependent pathways of cell death [53].

Identified mechanisms that can explain tumor selectivity of virus replication and oncolysis in non-permissive hosts involve: (i) defects in activation of anti-viral signaling pathways; (ii) defects in type I IFN signaling pathways; (iii) defects in apoptotic pathways; and (iv) activation of Ras signaling and expression of Rac1 protein [54].

In permissive hosts, namely avian cells, NDV has developed a frameshift variant of the viral phosphoprotein P to escape type I interferon mediated anti-viral responses [55]. This V protein, which targets STAT1 for degradation and leads to inhibition of an IFN-β response, is—fortunately for us humans—species-specific, and functions only in avian cells.

For therapy with oncolytic viruses (OVs), the anti-viral immune response limits OV replication and spread in situ. An initial period of vigorous OV multiplication and lytic activity seems necessary for development of subsequent anti-tumor immunity. Anti-viral T helper cells, however, could also have an interesting effect upon recruitment of DCs to the OV containing vaccination site [56]. The use of histone deacetylase inhibitors (HDAC) has been proposed as a means of overcoming barriers to oncolytic virotherapy [57].

### 8.3. Immunogenic Cell Death (ICD)

An important new paradigm of OV-mediated immunotherapy is the concept of ICD. Classical physiological apoptosis is non-immunogenic. It is characterized by membrane integrity, cell shrinkage, membrane blebbing, release of apoptotic bodies, nuclear condensation and DNA fragmentation. In contrast, immunogenic apoptosis induced by NDV involves immunogenic apoptosis with translocation to the plasma membrane of calreticulin (Ecto-CRT), heat shock proteins (HSPs) and the viral proteins HN and F. This process is then followed by necrosis with the release of cytokines (type I IFNs, TNFα) and chemokines (RANTES, IP-10). PAMPs, such as foreign viral RNA in the cytoplasm, recognized by RIG-I receptors [58], and HN at the cell surface recognized by NK cells via NKp46 receptors [59] contribute to this process, as does the release of damage-associated molecular patterns (DAMPs), such as HMGB1 [60].

### 8.4. Immune Response

A consequence of ICD is the induction of post-oncolytic anti-tumor immunity. Innate immunity activation involves NK cells, monocytes, macrophages and DCs, including plasmacytoid DCs. This leads to upregulation of TRAIL on NK cells and monocytes, to induction of cytotoxicity by NK cells, to release of nitric oxide (NO) and TNF-α from macrophages, and to the secretion of high amounts of type I IFNs by monocytes, PDCs and DCs [54].

NDV-induced IFN-α serves as a link between innate and adaptive immunity. In normal cells of non-permissive hosts (rodents, humans), signaling via RIG-I (intracellular recognition of viral RNA) and amplification via an IFN induced feedback loop by membrane-expressed type I IFN receptor (IFNRA) [61] leads to an effective interferon response, induction of anti-viral gene expression and prevention of NDV replication. NDV and NDV-induced IFNα is involved in polarization of DCs towards DC1, a genetic programming process that only takes 18 h and involves 24 transcription factors leading to upregulation of 779 genes [62]. Interaction of naïve T cells with such polarized antigen-presenting cells (APCs) then leads to polarization of T cells towards Th1. IFN-α also enhances cross-presentation in human DCs by modulating antigen survival, endocytic routing, and processing [63].

Infection of human tumor cells by NDV (*Ulster*) was shown to lead to upregulation of MHC molecules and of the adhesion molecules ICAM-1 and LFA-3 [64]. ATV-NDV vaccine cells were demonstrated to have an increased capacity for T cell adhesion and co-stimulation and to break T-cell tolerance [65]. These are all good pre-requisites for the re-activation of TAA-specific memory T cells. Such re-activation has been proposed as a main mechanism of the function of the vaccine ATV-NDV [36].

The induction of tumor-specific immune memory by intra-tumoral inoculation of NDV was demonstrated to be dependent on CD8 T cells and on RAG2 [60].

### 8.5. Potential to Break Therapy Resistancies

Apart from their capacity to replicate in resting cells such as tumor stem cells, NDV shows selectivity for apoptosis-resistant cells [66], hypoxic cancer cells [67] and interferon-resistant tumor cells [65].

Resistance to immunotherapy can also become affected by NDV infection. This was shown by breaking T-cell tolerance [68,69], and also by breaking resistance to immune checkpoint blockade immunotherapy [70].

## 9. Why Has It Taken So Long?

As is often the case with innovation, at the beginning, it is often met by skepticism. This seems particularly true in the field of oncology, where oncologists are trained to always apply the latest standard therapy. CT and RT are often considered as the latest gold standard. Now, if pioneers come along, for instance from cell biology (Cassel) or immunology (Schirrmacher) with a different view or vision, and make claims that things could be done in a different, and perhaps better, way, it is clear that this provokes reactions from the oncology establishment. The more the pioneer insists and continues, the more hurdles and roadblocks are put up to make them stop and fail.

Thus, the question is not only to have a good idea for future progress in cancer therapy, but a great deal of perseverance is needed to continue, especially when there is lack of industrial and financial support, and of scientific recognition, at times where such topics are not popular. For Cassel, it was the period of the 1960s and 1970s, and for Schirrmacher, the period of the 1980s and 1990s. Progress in NDV research and application could have been much faster if there had been support instead of resistance and prejudice. Considering the positive clinical results summarized in this review, patients could have profited from NDV much earlier.

The good news, however, is that the concept of using NDV for the improvements of cancer therapy has survived over five decades. Not only the ideas of viral oncolysis and post-oncolytic immune responses made it survive, but also its high safety profile.

Two other OVs have been approved for clinical application. H101 was the first oncolytic virus approved for cancer treatment in China, in 2006 [71]. H101 is an *E1B* gene deleted *Adenovirus*. In 2015, the FDA approved T-VEC, a *GM-CSF* gene-modified *Herpes Simplex Virus* (HSV), for application in advanced melanoma patients. T-VEC improved durable response rates [72]. The combination of T-VEC with the immune checkpoint blocking antibody Ipilimumab appeared to further increase its efficacy [73].

## 10. Summary

Table 1 presents an overview of 50 years of research on NDV. Basic research and clinical application are shown in parallel. Pioneering studies were performed in the 1960s and 1970s. Then, came a gap, in which the interest of cancer research scientists was greater in the development of cytostatic drugs and in the study of oncogenes and tumor suppressor genes. A revival of interest in OVs and NDV occurred in the 1990s and 2000s [74].

When used as a single agent, NDV has been applied in animal models and/or cancer patients by different routes (intra-tumoral, locoregional or systemic) and used over a large dose range. Specific vaccines, including NDV, were either viral oncolysates, live cell tumor vaccines or DC vaccines pulsed with viral oncolysate.

The types of human cancer that were treated are highlighted in this review. They include 10 different types: GBM, Gastrointestinal carcinoma (Ca), CRC, breast Ca, ovarian Ca, HNSCC, renal cell Ca, prostate Ca, melanoma and acute leukemia.

Fifty years of study on oncolytic NDV-enhanced immunotherapy of cancer is a long time. In spite of the many different applications of NDV, of different strains, treatment modalities and cancer types, there were no serious adverse events. It can, thus, be concluded that NDV has a very high safety profile for clinical application.

It is my hope that this biologic agent, either wildtype or recombinant, will further increase its impact in the coming decades for the benefit of cancer patients.

## Figures and Tables

**Table 1:** Fifty years of research on NDV.

Time Period	Basic Research	Clinical Application	References
1960s	viral oncolysis, post-oncol immunity	single case observations	[1,2,3]
1970s	studies on mechanisms; viral oncolysate	post-op vaccination studies	[5,6,7,8,9,19]
1980s	ATV-NDV live cell vaccine	vaccination with viral oncolysates	[14,20,21,22,23,24]
1990s	genome sequencing, molecular studies	phase I–II studies (ATV-NDV)	[27,28,30,32,33]
	immune cell activation mechanisms	vaccination by inhalation	[11,41,42,51]
2000s	recombinant improved NDV strains	phase II/III studies	[31,34,35,39,40]
	-	systemic dose-escalation study	[17,18,44,46,53]
2010s	ICD mechanisms	VOL-DC, compassionate use studies	[36,37,38,48,60,61,62,68,75]
	high quality (GMP) virus production	-	[41,42,43,60,66,67,70]
